# Nonlinear dimensionality reduction methods for synthetic biology biobricks’ visualization

**DOI:** 10.1186/s12859-017-1484-4

**Published:** 2017-01-19

**Authors:** Jiaoyun Yang, Haipeng Wang, Huitong Ding, Ning An, Gil Alterovitz

**Affiliations:** 1 School of Computer and Information, Hefei University of Technology, Tunxi Road, Hefei, 230009 China; 2 Harvard Medical School, Boston Children’s Hospital, Boston, 02115 MA USA

**Keywords:** Visualization, Synthetic biology, Biobricks, Dimensionality reduction, Edit distance

## Abstract

**Background:**

Visualizing data by dimensionality reduction is an important strategy in Bioinformatics, which could help to discover hidden data properties and detect data quality issues, e.g. data noise, inappropriately labeled data, etc. As crowdsourcing-based synthetic biology databases face similar data quality issues, we propose to visualize biobricks to tackle them. However, existing dimensionality reduction methods could not be directly applied on biobricks datasets. Hereby, we use normalized edit distance to enhance dimensionality reduction methods, including Isomap and Laplacian Eigenmaps.

**Results:**

By extracting biobricks from synthetic biology database Registry of Standard Biological Parts, six combinations of various types of biobricks are tested. The visualization graphs illustrate discriminated biobricks and inappropriately labeled biobricks. Clustering algorithm K-means is adopted to quantify the reduction results. The average clustering accuracy for Isomap and Laplacian Eigenmaps are 0.857 and 0.844, respectively. Besides, Laplacian Eigenmaps is 5 times faster than Isomap, and its visualization graph is more concentrated to discriminate biobricks.

**Conclusions:**

By combining normalized edit distance with Isomap and Laplacian Eigenmaps, synthetic biology biobircks are successfully visualized in two dimensional space. Various types of biobricks could be discriminated and inappropriately labeled biobricks could be determined, which could help to assess crowdsourcing-based synthetic biology databases’ quality, and make biobricks selection.

**Electronic supplementary material:**

The online version of this article (doi:10.1186/s12859-017-1484-4) contains supplementary material, which is available to authorized users.

## Background

In synthetic biology, one of the most important tasks is to assemble various standardized gene segments, i.e. biobricks, to form artificial biological devices with specific functions [1, 2]. Therefore, the establishment of biobricks database appears to be particularly important. Due to the rapid development of this area, numerous amount of biobricks have been generated, e.g. about 30,000 biobricks in Registry of Standard Biological Parts (http://parts.igem.org/Main_Page) [3]. This brings several challenges for this domain. One is that the database is constructed by crowdsourcing strategy [4], which means anyone could contribute to the database, hence it could not guarantee biobricks’ quality. Another one is that so many biobricks make it hard to choose one for constructing devices.

In order to overcome above issues, analytical methodology is urgently needed to make quality assessment and interpretation. An efficient method is to reduce the dimensions of biobricks and visualize them in two or three dimensional spaces, then some patterns may emerge, e.g. similar data would flock together to become clusters, and they could be easily observed in the graph. This could help to get a first impression of properties or the quality of the database. Dimensionality reduction for visualization has been successfully applied in many areas, e.g. microarray data analysis, etc. [5, 6]. In the visualized graph, a point denotes an item, e.g. gene, biorbrick, etc. The distance between points usually represents the similarity. Hence, the closer two biobricks are in the graph, the more similarity they have. It has been showed that similar genes have similar functions or structures [7]. There are various types of biobricks, corresponding to different functions, e.g. promoters initiate transcription of a particular gene, primers are used as a starting point for PCR amplification or sequencing, etc. If a biobrick is visualized among some biobricks with different types, it may be marked with inappropriate types. Besides, when users select a biobrick in the graph, they could also find other biobricks with similar functions, which could help to determine an appropriate biobrick to use.

There are mainly two categories of methods for dimensionality reduction. One is feature selection, which is to select a subset of features to represent the whole samples [8, 9]. If applied here, it would be choosing two or three gene sites as representative of the biobricks. As biobricks are gene segments with length ranging from several hundred to several thousand, only using two or three gene sites to denote the whole segments would lose most of the information and is unreliable.

The other category of methods for dimensionality reduction is feature extraction, which builds derived features by mapping features from high dimensional space to low dimensional space. There are essential difference between feature selection and feature extraction. The former one just selects a subset of original features, while the latter one needs to generate new features, which are totally different from original features. Therefore, feature extraction is more suitable for biobricks’ dimensionality reduction than feature selection.

Feature extraction methods could be grouped into two categories, linear dimensionality reduction and nonlinear dimensionality reduction [10]. The features derived by linear dimensionality reduction could be regarded as linear combinations of original features. A classical linear dimensionality reduction method is principal component analysis (PCA), which first constructs data covariance (or correlation) matrix, and then applies eigenvalue decomposition to obtain mapped results [11, 12]. As an unsupervised learning method, PCA is widely used to deal with large scale unlabeled data. However an issue emerges when applying PCA. Biobricks are gene segments with various lengths, while data covariance matrix consists of covariance of two samples and requires the identical dimension of various samples. Therefore, it is impractical to construct covariance matrix based on these biobricks. Multi-Dimensional Scaling (MDS) and its improved linear methods first construct a distance matrix on the dataset and then embed the data in low dimensions by eigendecomposition [13]. Current distance matrix is evaluated in Euclidean space, which requires to conduct numerical operations on data with identical dimension. Biobricks are represented as sequences with various lengths in computer, besides numerical operation on the characters in biobricks could not represent the similarity between biobricks, therefore current distance matrix could not be applied on biobricks.

Nonlinear dimensionality reduction is mainly based on manifold learning and could handle data’s nonlinear property. One kind of these methods are established on the extension of linear methods. For example, kernel PCA extends PCA by applying a kernel function to the original data and then performing PCA process [14]. Isomap is an extension of MDS and tries to maintain the intrinsic geometry of by adopting an approximation of the geodesic distance on the manifold, where the geodesic distance is calculated by summing the Euclidean distances along the shortest path between two nodes [15]. Since linear methods are not suitable for processing biobricks and this kind of methods still involve linear methods, they are also not the right choice for handling biobricks.

Another kind of nonlinear dimensionality reduction methods adopt various strategies to capture the geometry structure and apply eigendecomposition to maintain the structure in a lower-dimensional embedding of the data. The classical methods include Local Linear Embedding (LLE), Laplacian Eigenmaps, etc. LLE assumes each sample could be represented as the linear combination of its local neighbor samples, and tries to find an embedding that could preserve the local geometry in the neighborhood of each data point [16]. Some methods are proposed to improve LLE’s quality, such as Hessian Locally-Linear Embedding (HLLE) [17], Modified Locally-Linear Embedding (MLLE) [18], etc. However, when applying these methods here, an issue emerges that it is usually hard to use a combination of gene segments to denote another segment, especially when the lengths are not identical. Laplacian Eigenmaps is according to the assumption that the Laplacian of the graph obtained from the data points may be reviewed as an approximation to the Laplace-Beltrami operator defined on the manifold [19, 20]. This method regards each data point as a node in a graph, and the connection of nodes is based on k-nearest neighbor strategy. It needs to calculate the Euclidean distance to construct the graph, therefore it faces the issue that Euclidean distance is not applicable for gene segments.

From the above analysis, we can see that current dimensionality reduction methods could not be directly applied to biobricks, and it is mostly because of the coordinate calculation for various purposes. Among these purposes, there is a specific one that coordinate calculation is used to measure the similarities of samples and help to find the neighborhood, including MDS, Isomap, Laplacian Eigenmaps. We could find alternative methods for biobricks’ similarity calculation. Actually edit distance is a widely used measurement for gene similarity, and it is equal to the minimum number of operations required to transform one gene sequence into the other. Therefore, edit distance could be combined with this specific group of method to reduce biobricks’ dimensionality.

In this paper, we propose to combine edit distance with dimensionality reduction methods for biobricks’ visualization. By adopting normalized edit distance to construct similarity matrix, both Isomap and Laplacian Eigenmaps successfully accomplish biobricks’ dimensionality reduction, and visualize the dataset derived from Registry of Standard Biological Parts. Besides, Laplacian Eigenmaps is 5 times faster than Isomap, and its visualization graph is more concentrated to discriminate biobricks. Furthermore, clustering algorithm K-means is applied on the dimensionality reduction results to quantify the dimensionality reduction performance. The average clustering accuracy for Isomap and Laplacian Eigenmaps are 0.857 and 0.844, respectively, which indicate that the proposed dimensionality reduction methods could preserve the underlying structure of biobricks, and the visualization results could reflect the relationships among biorbicks.

The rest of this paper is organized as follows. We first formulate the dimensionality reduction problem for synthetic biology, and then describe the edit distance and how to combine edit distance with Isomap and Laplacian Eigenmaps. After that, the dataset used in this paper will be introduced and the visualization and clustering results will be illustrated in the results and discussion section. In the last, we summarize the paper.

## Methods

In this section, we first formulate the dimensionality reduction problem. Then we introduce the normalized edit distance and how to combine it with dimensionality reduction methods.

### Problem formulation

Let *X*={*x*_1_,*x*_2_,…,*x*_*n*_} be a set of DNA sequences defined on a finite alphabet *Σ*={*A*,*T*,*C*,*G*}, where $\phantom {\dot {i}\!}x_{i}=s_{1}s_{2}\ldots s_{|x_{i}|}$ represents a biobrick with length |*x*_*i*_|.

The dimensionality reduction problem for synthetic biology is to find a vector set *Y*={*y*_1_,*y*_2_,…,*y*_*n*_}, where *y*_*i*_ is the reduction result of *x*_*i*_, and these vectors satisfy: 1) ∀*i* (1≤*i*≤*n*), *y*_*i*_ is a *k*-dimension vector that could be represented in Euclidean Space; 2) Vectors in *Y* should maintain the underlying structure among biobircks in *X*.

For the first constraint, the value of *k* is usually 2 or 3, so that the vector could be visualized in a 2-D or 3-D space. For the second constraint, the most common underlying structure among the original dataset is manifold. In order to capture the structure, various algorithms set different optimization functions, and convert the problem into an optimization problem to achieve the reduction results. Another important difference among these algorithms is the way of constructing similarity matrix. In this paper, we focus on Isomap and Laplacian Eigenmaps, and the detailed process will be discussed in the next section.

### Normalized edit distance

Assume *x*_*i*_ and *x*_*j*_ are two biobricks in *X*, and their lengths are |*x*_*i*_| and |*x*_*j*_|, respectively.

The edit distance *d*(*x*_*i*_,*x*_*j*_) is defined as the minimum summation of edit operations’ weight that transforms *x*_*i*_ into *x*_*j*_, where the edit operation could be insertion, deletion, substitution, etc. The classical algorithm for edit distance calculation is dynamic programming, which recursively constructs a score matrix *T* with size (|*x*_*i*_|+1)∗(|*x*_*j*_|+1). In matrix *T*, *T*[*p*_*i*_,*p*_*j*_] contains the edit distance of prefixes *x*_*i*_[1…*p*_*i*_] and *x*_*j*_[1…*p*_*j*_]. If let *w*_*ins*_, *w*_*del*_, *w*_*sub*_, *w*_*mat*_ denote the weight of insertion, deletion, substitution and match operation, the recursive formula is as follows:

1 $$ T\left[p_{i},p_{j}\right]=\min \left\{\begin{array}{ll} T\left[p_{i}-1,p_{j}\right]+w_{del}\\ T\left[p_{i},p_{j}-1\right]+w_{ins}\\ T\left[p_{i}-1,p_{j}-1\right]+w_{sub}\\ \verb+ + \textrm{if \(x_{i}\left[p_{i}\right]\neq x_{j}\left[p_{j}\right]\)}\\ T\left[p_{i}-1,p_{j}-1\right]+w_{mat}\\ \verb+ + \textrm{if \(x_{i}\left[p_{i}\right]= x_{j}\left[p_{j}\right]\)}\\ \end{array} \right.   $$

**Fig. 1:**
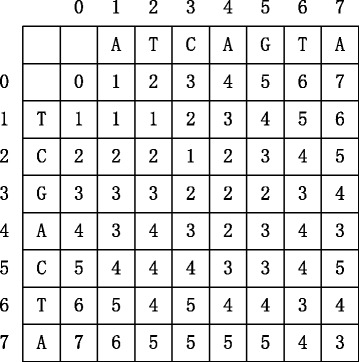
Dynamic table *T* for calculating the edit distance between DNA sequence ATCAGTA and TCGACTA. The optimal edit distance is 3, i.e. the value in cell *T*[7,7]

For example, if let *w*_*del*_, *w*_*ins*_, *w*_*sub*_ be equal to 1, and *w*_*mat*_ be equal to 0. Figure 1 illustrates the dynamic table *T* for DNA sequence ATCAGTA and TCGACTA, where the value is calculated based on Eq. 1. The edit distance of these two sequences is 3, i.e. the value in cell *T*[7,7]. It denotes that at least 3 edit operations are needed to transform ATCAGTA into TCGACTA.

The length of bioricks varies a lot, ranging from several hundred to several thousand. Thus there are huge differences between edit distances of various biobricks. For example, the edit distance of two biobricks with length 1000 and 800 is at least 200, while the edit distance of two biobricks with length 200 and 100 is at most 200. The former distance is larger than the latter one. However, we could not draw the conclusion that the former two biobricks are less similar than the latter two biobricks. Therefore, we adopt a normalized distance to represent the edit distance, which is defined as follow:

2 $$ n\_d(x_{i},x_{j})=\frac{d(x_{i},x_{j})}{max(length(x_{i}), length(x_{j}))}  $$

Where *d*(*x*_*i*_,*x*_*j*_) represents the edit distance of *x*_*i*_ and *x*_*j*_, and *m* *a* *x*(*l* *e* *n* *g* *t* *h*(*x*_*i*_),*l* *e* *n* *g* *t* *h*(*x*_*j*_)) denotes the maximum value of the length of *x*_*i*_ and *x*_*j*_.

Based on *n*_*d*(*x*_*i*_,*x*_*j*_), a matrix *M* could be constructed as Eq. 3, where *M*_*ij*_ represents the normalized edit distance of *x*_*i*_ and *x*_*j*_, i.e. *M*_*ij*_=*n*_*d*(*x*_*i*_,*x*_*j*_).

3 $$ M= \left(\begin{array}{cccc} M_{11} & M_{12} &\dots & M_{1n} \\ M_{21} & M_{22} &\dots & M_{2n} \\ \vdots & \vdots &\ddots & \vdots \\ M_{n1} & M_{n2} &\dots & M_{nn} \\ \end{array} \right)   $$

### Isomap with normalized edit distance

Isomap algorithm maintains the manifold structure by optimizing the following function:

4 $$ \min \left(\sum_{i=1}^{n}\sum_{j=1}^{n} \left({S_{ij}} - ||y_{i}-y_{j}||\right)^{2}\right)^{-\frac{1}{2}}  $$

Where *S*_*ij*_ is the similarity of *x*_*i*_ and *x*_*j*_, and *y*_*i*_ and *y*_*j*_ denote the reduction results of *x*_*i*_ and *x*_*j*_, respectively.

The dimensionality reduction problem is converted to an optimization problem, and Isomap solves it through three main steps.

The first step is to establish the neighborhood graph, which could be constructed based on matrix *M*. Each node in the graph G represents a biobrick, and if *x*_*j*_ is one of the K nearest neighbors of *x*_*i*_, there is an edge to connect *x*_*i*_ and *x*_*j*_ by assigning weight *M*_*ij*_. Otherwise, the weight of *x*_*i*_ and *x*_*j*_ is equal to infinity. In other words, Isomap reconstructs matrix *M* by replacing the value *M*_*ij*_ by infinity if *x*_*j*_ is not one of the K nearest neighbors of *x*_*i*_.

The second step is to calculate the shortest path of *x*_*i*_ and *x*_*j*_ to approximate the geodesic distance, and the shortest path distance is used to represent the similarity of *x*_*i*_ and *x*_*j*_. Here we apply *S*_*ij*_ to denote this similarity. There have been many successful algorithms to find the shortest path, among which Floyd’s algorithm is a classical one. It performs the following process: for each value *k*=1,2,…,*n* in turn, replace the value of *S*_*ij*_ by min{*S*_*ij*_,*S*_*ik*_+*S*_*kj*_}, and the initial value of *S*_*ij*_ is the same as the reconstruction matrix in the first step. After achieving matrix *S*, we should square each value in *S* before processing the next step.

The third step is to construct d-dimension embedding, which is done by the eigendecomposition of matrix *D*. *D* is constructed based on Eq. 5.

5 $$ {G_{ij}}=-\frac{1}{2}\left({S_{ij}}-\frac{1}{n}D_{i}-\frac{1}{n}D_{j}+\frac{1}{n^{2}}D_{i}D_{j}\right)   $$

Where *D*_*i*_ is computed according to Eq. 6.

6 $$ D_{i}=\sum_{1\leq j\leq n} {S_{ij}}   $$

Assume *λ*_*p*_ is the *p*−*t* *h* eigenvalue (in decreasing order) of matrix *D*, and $v_{p}^{i}$ is the *i*−*t* *h* component of the *p*−*t* *h* eigenvector. Then the *p*−*t* *h* component of the embedding results *y*_*i*_ for sample *x*_*i*_ is equal to $\sqrt {\lambda _{p}}v_{p}^{i}$.

### Laplacian eigenmaps with normalized edit distance

Different from Isomap algorithm, Laplacian Eigenmaps employs the following Eq. 4 as the optimization function.

7 $$ min \sum_{i=1}^{n}\sum_{j=1}^{n} ||y_{i} - y_{j}||^{2}{S_{ij}}   $$

Where *S*_*ij*_ is the similarity of *x*_*i*_ and *x*_*j*_, and *y*_*i*_ and *y*_*j*_ represent the reduction results of *x*_*i*_ and *x*_*j*_, respectively.

Note that the similarity matrix *S* here is different from Isomap’s similarity matrix *S*. Laplacian Eigenmaps applies a kernel function on matrix *M* to achieve *S*. A widely used kernel function is Gaussian kernel, which is defined as Eq. 8.

8 $$ f(M_{ij})= e^{-\frac{M_{ij}^{2}}{2\sigma^{2}}}   $$

After constructing the similarity matrix *S*, Laplacian Eigenmaps solves the problem by applying eigendecomposition to matrix *G*, where *G*=*D*−*S* and *D* is a diagonal matrix with the values *D*_1_,*D*_2_,…,*D*_*n*_ on the diagonal. *D*_*i*_ could be calculated based on 6. The final embedding result *y*_*i*_ consists of the *i*−*t* *h* component of the first *k* eigenvectors.

**Fig. 2:**
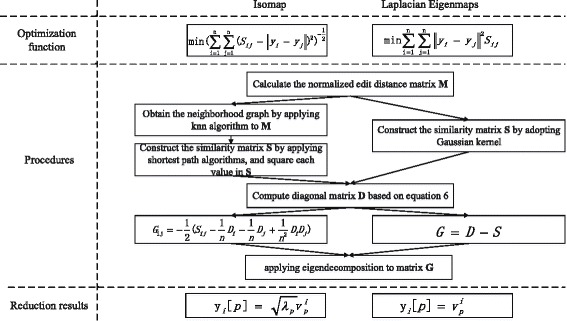
Comparison of Isomap and Laplacian Eigenmaps. The comparison is performed in terms of optimization functions, procedures and reduction results, where *y*_*i*_[*p*] denotes the *p*−*t* *h* component of *y*_*i*_

Figure 2 illustrates the comparison of Isomap and Laplacian Eigenmaps in terms of optimization function, procedures and reduction results. Both algorithms share some steps, such as calculating the normalized edit distance matrix *M*, computing the diagonal matrix *D*, and applying eigendecomposition to matrix *G*. The main differences are the way of constructing similarity matrix *S*, matrix *G*, and achieving reduction results. The first difference is because Isomap employs geodesic distance to denote the similarity, and the latter two differences are due to the diverse optimization functions.

## Results and discussion

In this section, the synthetic biology dataset is first introduced, and then we illustrate the dimensionality reduction results of employing Isomap and Laplacian Eigenmaps to the dataset. Besides, the clustering algorithm is adopted on the dimensionality reduction results to validate the performance.

### Datasets and implementation details

The dataset is obtained from ‘Registry of Standard Biological Parts’ (http://parts.igem.org/), which is a publicly available synthetic biology database for storing biobricks. There are mainly 26 categories of biobricks. The category information is achieved from each part’s XML files provided by the registry. According to the official site description, these various categories of biobricks belong to 11 types, which means some types have several subtypes. Without loss of generality, four different types of biobricks are selected to validate the algorithms, including protein generators, protein domains, Ribosomal Binding Site (RBS), primers. The number of these four biobricks are 500, 500, 300, and 500, respectively. There are only 300 RBS in the database, so these numbers are not equal. More experiments about other types of biobricks are included in Additional file 1.

These four different types of biobricks correspond to various functions. Protein generators are parts or devices used for generating proteins. Protein domains are conserved parts of given protein sequences and could make up a protein coding sequence with the rest of protein chains. A RBS is a sequence of nucleotides upstream of the start codon of an mRNA transcript. A primer is a short single-stranded DNA sequences used as a starting point for PCR amplification or sequencing.

The presented methods were implemented in python 2.7.11 and Matlab 8.4.0 (R2014b). The python-package, levenshtein, was used to compute the edit distance of each two genes. The dimensionality reduction algorithms Isomap and Laplacian Eigenmaps were implemented in Matlab. In the final of routine performing, we evaluated the accuracy by results of K-means, which was implemented in Matlab. Isomap needs to adopt knn algorithm, the parameter K is set to 30% of the dataset size. Laplacian Eigenmaps applies Gaussian kernel, and the parameter *σ* is set to 0.3.

### Dimensionality reduction results

**Fig. 3:**
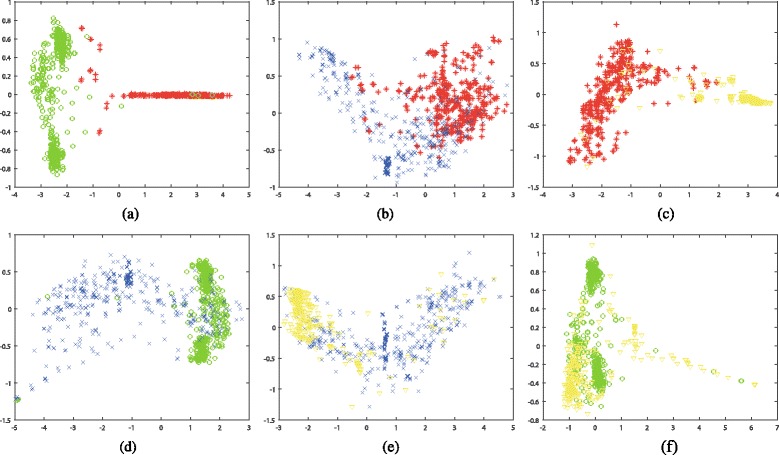
2-D visualization for the dimensionality reduction results by applying Isomap algorithm. The datasets are composed of various combinations of Protein generators, Primers, Protein domains, and RBS, where R, G, B, Y denote red color, green color, blue color, and yellow color, respectively. **a** Protein generators (R) and Primers (G), **b** Protein generators (R) and Protein domains (B), **c** Protein generators (R) and RBS (Y), **d** Primers (G) and Protein domains (B), **e** Protein domains (B) and RBS (Y) (f) Primers (G) and RBS (Y), **f** Primers (G) and RBS (Y)

**Fig. 4:**
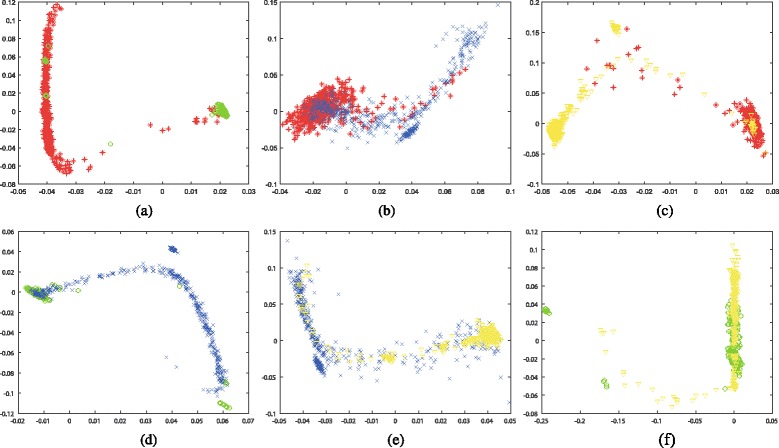
2-D visualization for the dimensionality reduction results by applying Laplacian Eigenmaps algorithm. The datasets are composed of various combinations of Protein generators, Primers, Protein domains, and RBS, where R, G, B, Y denote red color, green color, blue color, and yellow color, respectively. **a** Protein generators (R) and Primers (G), **b** Protein generators (R) and Protein domains (B), **c** Protein generators (R) and RBS (Y), **d** Primers (G) and Protein domains (B), **e** Protein domains (B) and RBS (Y), **f** Primers (G) and RBS (Y)

We first conduct dimensionality reduction on various combination of these four types of biobricks with Isomap and Laplacian Eigenmaps algorithms. Thus, these various types of biobricks with different lengths are reduced to two dimension vectors. Then, these reduction results are visualized in graphs. Figures 3 and 4 illustrate the 2-D visualization results for Isomap and Laplacian Eigenmaps, respectively. Each subfigure shows the visualization of two different biobricks, where protein generators, primers, protein domains, and RBS are marked with red color, green color, blue color, and yellow color, respectively.

The visualization results demonstrate that various combinations of these biobricks could be separated after dimensionality reduction. The distribution of these combinations varies a lot. Generally speaking, the results generated by Laplacian Eigenmaps are more concentrated than that of Isomap. This may because that Isomap adopts shortest path algorithm to calculate the similarity, while Laplacian Eigenmaps applies Gaussian kernel on the similarity matrix, and some similarities may become zero after this process, which makes the points in the graph more concentrated. These findings could also be found on other types of biobricks [see Additional file 1: Figures S1 and S2].

Among all the combinations, the combination of protein generators and primers achieves the best discrimination for both Isomap and Laplacian Eigenmaps, which means protein generators and primers have the most dissimilarity among all these six combinations. Combinations of protein generators and RBS, primers and protein domains also obtain promising discrimination, while combinations of protein generators and protein domains, primers and RBS are not easy to distinguish, which means many of these biobricks share some similarities.

In addition, we could find that even for a finite type of biobrick, the visualization may present some clusters. For example, there are three obvious clusters for primers in the subfigure (a), (d) and (f) of Figs. 3 and 4 (marked with green color). These clusters denote different types of primers. One type is inter-strain nested primer. One type is used for genomic integration and expression of Green fluorescent protein under the control of various promoters. Actually these biobricks might not be appropriate to be marked as primer according to their function. In Figs. 3(a) and 4(a), they are closer to protein generators, even mixed in them.

Except for inappropriately labeled primers, there are also some other inappropriately labeled bioricks. Figures 3 and 4 shows some protein generators are closer to primers or RBS. Actually these protein generators are only composed of promoter and RBS, e.g. BBa_K143050, etc., and they do not contain any coding sequences. Therefore, they might not be suitable to be labeled with protein generators. In Figs. 3(c) and 4(c), some RBS are mixed with protein generators. When checking the biobricks’ documents, some of them have coding sequences, e.g. BBa_K079013, etc., and some of them do not have any explanations, e.g. BBa_K294120, etc. This demonstrates that the visualization could help to determine whether the biobricks are appropriately labeled. Besides, similar biobricks have close distance in the graph. Users could find a set of biobricks for a specific function in the graph and choose the best one for their purpose.

**Fig. 5:**
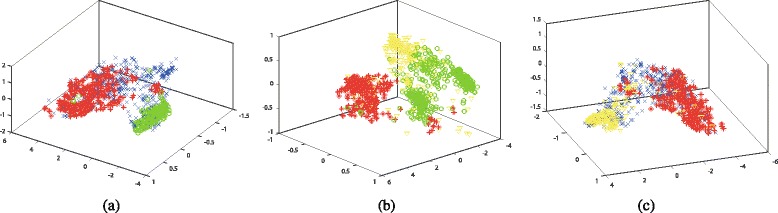
3-D visualization for the dimensionality reduction results by applying Isomap algorithm. The datasets are composed of various combinations of Protein generators, Primers, Protein domains, and RBS, where R, G, B, Y denote red color, green color, blue color, and yellow color, respectively. **a** Protein generators (R), Primers (G) and Protein domains (B). **b** Protein generators (R), Primers (G) and RBS (Y). **c** Protein generators (R), Protein domains (B) and RBS (Y)

**Fig. 6:**
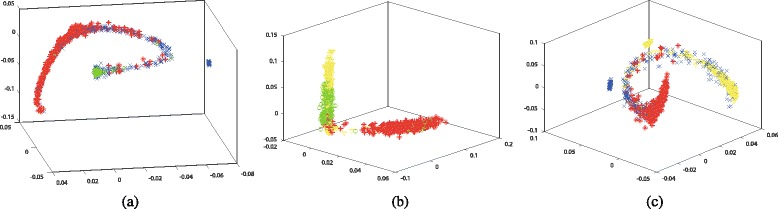
3-D visualization for the dimensionality reduction results by applying Laplacian Eigenmaps algorithm. The datasets are composed of various combinations of Protein generators, Primers, Protein domains, and RBS, where R, G, B, Y denote red color, green color, blue color, and yellow color, respectively. **a** Protein generators (R), Primers (G) and Protein domains (B), **b** Protein generators (R), Primers (G) and RBS (Y), **c** Protein generators (R), Protein domains (B) and RBS (Y)

Furthermore, we tested the 3-D visualization results of Isomap and Laplacian Eigenmaps by mixing any three types of biobricks together. Figures 5 and 6 demonstrate the results. 3-D graphs could be viewed from any angles, however we could only show them in a particular angle in the paper. Discriminated biobricks still emerge based on various types. Besides, there are also clusters like 2-D graphs. For example, there are still three clusters of primers corresponding to different functions. Besides, the distribution of inappropriately labeled biobricks is similar as that in 2-D graphs.

### Time performance

We also test the time performance of Isomap and Laplacian Eigenmaps algorithms. Both algorithms need to calculate the edit distance, thus this calculation is performed independently, and is not included in this time comparison. Table 1 shows the results. Laplacian Eigenmaps consumes much less time than Isomap, at least 5 times faster. According to Fig. 2, the most different step for these two algorithms is to calculate the similarity matrix. Isomap needs to apply knn algorithm and shortest path algorithm to achieve the similarity matrix, while Laplacian Eigenmaps only applies Gaussian kernel on the edit distance matrix. knn algorithm and shorest path algorithm have larger time complexity than calculating Gaussian kernel, and this results in much larger time consumption of Isomap than Laplacian Eigenmaps. For the combinations containing RBS, both algorithms would cost shorter time than other combinations, this is because the size of RBS is smaller than other biobricks. The time of Isomap decreases larger than that of Laplacian Eigenmaps, which means the time performance of Isomap is more sensitive about the data size than that of Laplacian Eigenmaps.

**Table 1:** Comparison of Isomap and Laplacian Eigenmaps in terms of time consumption

	Isomap	LE
Protein Generators and Primer	7.0s	0.80 s
Protein Generators and Protein domains	6.75s	0.75s
Protein Generators and RBS	3.9s	0.73 s
Primer and Protein domains	6.8s	1.04s
Protein domains and RBS	3.8s	0.70 s
Primer and RBS	3.8s	0.73s

LE denotes Laplacian Eigenmaps

### Clustering validation

In order to quantify the dimensionality reduction results, we adopt a classical clustering algorithm, K-means, on the results to determine how well the combination of various types of biobricks could be discriminated.

K-mean is an unsupervised clustering algorithm to group samples into different clusters based on distances between samples [21]. It performs the following procedures.

1. Randomly select *K* samples as the initial centroids.
2. For each sample *i*, compute the distance between sample *i* and all the centroids, and find the centroid *k* with the smallest distance. Then assign sample *i* to cluster *k*.
3. Recompute the centroids for each cluster by averaging all the samples among this cluster.
4. If the centroids change compared with previous centroids, go to step 2.
5. End the algorithm.

Since the dimensionality reduction results are numerical vectors, we adopt Euclidean distance to measure the distance. The parameter K is set to 2 since there are two types of biobricks for each combination. Clustering accuracy is defined as Eq. 9, where *N* and *c*_*i*_ denote the number of samples and the number of samples that are correctly assigned to the *i*−*t* *h* cluster, respectively.

9 $$ Accuracy =\frac{\sum_{i=1}^{k} c_{i}}{N}   $$

Table 2 shows the clustering accuracies by applying K-means algorithm on the 2-D dimensionality reduction results generated by Isomap and Laplacian Eigenmaps. Protein generators and Primers achieve the best clustering accuracy, while Protein generators and Protein domains obtain the worst clustering accuracy, which are consistent with the visualization results. The clustering accuracy for Isomap is better than that of Laplacian Eigenmaps except for Protein domains and RBS, this may because Laplacian Eigenmaps applies Gaussian kernel to the distance matrix, and some distances become 0. Actually, this property also makes the visualization results more concentrated. The average accuracies of these six datasets are 0.857 and 0.844 for Isomap and Laplacian Eigenmaps, respectively.

**Table 2:** Clustering accuracy comparison of 2-D dimensionality reduction results by Isomap and Laplacian Eigenmaps

	Isomap	LE
Protein generators and primer	0.97	0.97
Protein generators and protein domains	0.77	0.72
Protein generators and RBS	0.95	0.92
Primer and protein domains	0.86	0.845
Protein domains and RBS	0.81	0.83
Primer and RBS	0.78	0.78

LE denotes Laplacian Eigenmaps

Table 3 demonstrates the clustering accuracies on the 3-D dimensionality reduction results obtained by Isomap and Laplacian Eigenmaps. The average accuracies of these datasets generated by Isomap and Laplacian Eigenmaps are 0.927 and 0.928, respectively, which validates the effectiveness of dimensionality reduction methods, and denotes that different types of biobricks could be easily separated after visualizing them in one graph. Clustering results on other types of biobricks could be found in Additional file 1: Table S1.

**Table 3:** Clustering accuracy comparison of 3-D dimensionality reduction results by Isomap and Laplacian Eigenmaps

	Isomap	Laplacian Eigenmaps
Protein generators, primers and protein domains	0.944	0.918
Protein generators, primers and RBS	0.915	0.955
Protein generators, protein domains and RBS	0.922	0.911

The average accuracy shows how well different types of biobricks could be separated. Isomap and Laplacian Eigenmaps differ a little on the accuracy. This difference is caused by the various ways of calculating similarity matrices. Isomap first applies knn algorithm to construct the neighbor graph, then adopts the shortest path algorithm to achieve the similarity matrix, while Laplacian Eigenmaps only applies Gaussian kernel on the edit distance matrix. After applying Gaussian kernel, some distances may become 0. This operation may cause information lost, however it could make the graph more concentrate to discriminate biobricks. Besides, Laplacian Eigenmaps is much faster than Isomap. Therefore, Laplacian Eigenmaps is more suitable for handling large size datasets.

Besides, classification validation is also conducted on these biobricks, and the results could be found in Additional file 1: Table S2.

## Conclusions

In this paper, we propose to combine normalized edit distance with Isomap and Laplacian Eigenmaps for biobricks’ dimensionality reduction and visualization. The visualization results illustrate that different types of biobricks could be easily distinguished by applying the proposed method, and some inappropriately labeled biobricks could be determined. Besides, K-means algorithm is adopted to quantify the dimensionality reduction results. The average clustering accuracy of six various combinations of biobricks are 0.857 and 0.844 for the proposed two algorithms, respectively. This validates that different types of biobricks could be separated in the visualized graph by applying the proposed dimensionality reduction methods. It also implies the visualization could help to assess the quality of biobricks in the crowdsourcing based synthetic biology database.

## Additional file

Additional file 1 This file contains more experiments on other types of biobricks. Besides, classification validation for the dimensionality reduction results are also included. These results are illustrated in two figures and two tables in the file. **Figure S1**: Dimensionality reduction results for various combinations of Plasmid backbones, Promoters, Terminators, Translational units, Protein generators, Primers by applying Isomap algorithm. **Figure S2**: Dimensionality reduction results for various combinations of Plasmid backbones, Promoters, Terminators, Translational units, Protein generators, Primers by applying Laplacian Eigenmaps algorithm. **Table S1**: Clustering accuracy comparison of dimensionality reduction results in **Figures S1** and **S2**. **Table S2**: Classification accuracy comparison of dimensionality reduction results by Isomap and Laplacian Eigenmaps. (PDF 123 kb)

## Abbreviations

LLE: Local linear embedding
LE: Laplacian Eigenmaps
MDS: Multi-dimensional scaling
PCR: Polymerase chain reaction
PCA: Principal component analysis
RBS: Ribosomal binding site
