# Supplementary material for: Nonlinear dimensionality reduction methods for synthetic biology biobricks’ visualization
Source: BMC Bioinformatics. 2017 Jan 19;18:47. doi: 10.1186/s12859-017-1484-4 (PMC5248484; doi:10.1186/s12859-017-1484-4)
Supplement: Additional file 1 — This file contains more experiments on other types of biobricks. Besides, classification validation for the dimensionality reduction results are also included. These results are illustrated in two figures and two tables in the file. Figure S1: Dimensionality reduction results for various combinations of Plasmid backbones, Promoters, Terminators, Translational units, Protein generators, Primers by applying Isomap algorithm. Figure S2: Dimensionality reduction results for various combinations of Plasmid backbones, Promoters, Terminators, Translational units, Protein generators, Primers by applying Laplacian Eigenmaps algorithm. Table S1: Clustering accuracy comparison of dimensionality reduction results in Figures S1 and S2. Table S2: Classification accuracy comparison of dimensionality reduction results by Isomap and Laplacian Eigenmaps. (PDF 123 kb) [file 12859_2017_1484_MOESM1_ESM.pdf]

Supplementary Material for

“Nonlinear Dimensionality Reduction Methods for Synthetic Biology Biobricks’ Visualization”

December 18, 2016

S1 Supplementary experiments on biobricks’ visualization

The datasets come from various combinations of plasmid backbones, promoters, terminators, translational units, protein generators and primers. The numbers of these different types of biobricks are 300, 500, 300, 500, 500, 500, respectively. Their functions are described below.

A plasmid backbone is defined as the plasmid sequence beginning with the biobrick suffix, and ending with the biobrick prefix. Promoters tend to recruit transcriptional machinery and lead to transcription of the downstream DNA sequence. Terminators usually occur at the end of a gene or operon mRNA and cause transcription to stop. Translational units begin at the site of translational initiation, and end at the site of translational termination. Protein generators are parts or devices used for generating proteins. A primer is a short single-stranded DNA sequence used as a starting point for PCR amplification or sequencing.

Figure S1 and Figure S2 illustrate the visualization of dimensionality reduction results achieved by Isomap and Laplacian Eigenmaps, where different types of biobricks are marked with different colors. We could find that clusters still emerge in the graphs, and each cluster could be regarded as a type of biobricks. The graphs achieved by LE are more concentrate than that obtained by Isomap, which is consistent with previous results.

Table S1 shows the clustering accuracies on the results in Figure S1 and Figure S2. The average accuracies for Isomap and Laplacian Eigenmaps are 0.883 and 0.878, respectively. The difference between these two methods is not quite large, which is also consistent with previous results. Among all the results, Plasmid backbones and Terminators achieve the highest clustering accuracy, which means they have the most dissimilarity.

Table S1: Clustering accuracy comparison of dimensionality reduction results in Figure S1 and Figure S2

	Isomap	Laplacian Eigenmaps
Plasmid backbones and Promoters	0.887	0.888
Plasmid backbones and Terminators	0.95	0.953
Plasmid backbones and Translational units	0.838	0.862
Promoters and Protein generators	0.896	0.884
Translational units and Primers	0.85	0.822
Translational units and Promoters	0.876	0.86

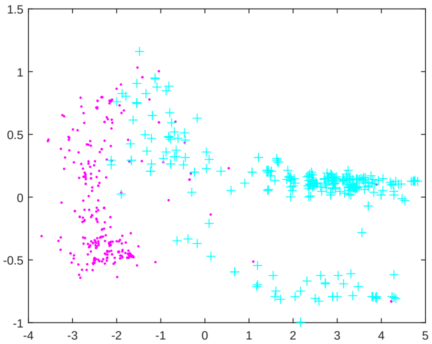

(a) Plasmid backbones (P) and Promoters (L)

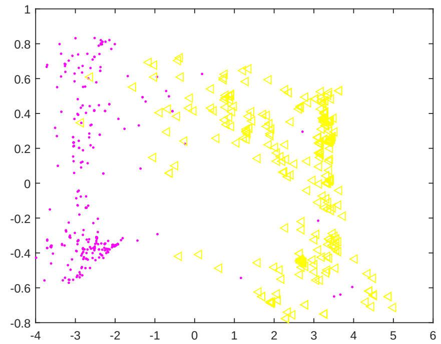

(b) Plasmid backbones (P) and Terminators (Y)

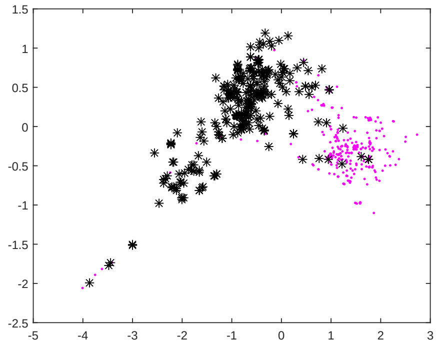

(c) Plasmid backbones (P) and Translational units (B)

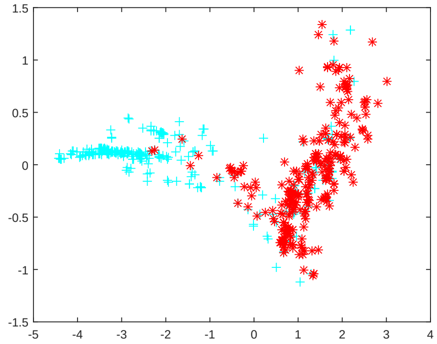

(d) Promoters (L) and Protein generators (R)

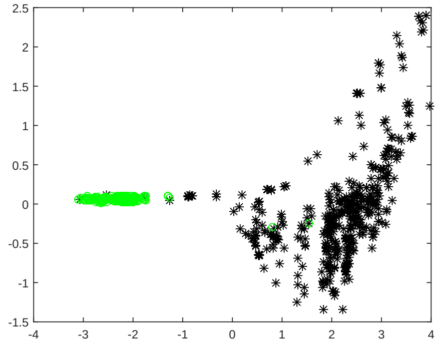

(e) Translational units (B) and Primers (G)

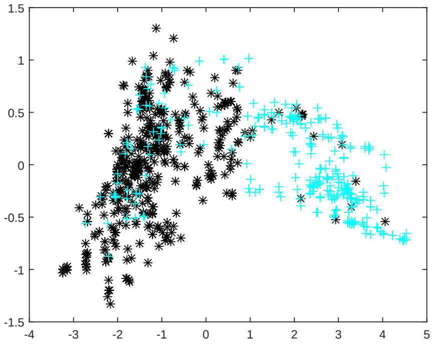

(f) Translational units (B) and Promoters (L)

Figure S1: Dimensionality reduction results for various combinations of Plasmid backbones, Promoters, Terminators, Translational units, Protein generators, Primers by applying Isomap algorithm, where these types of biobricks are denoted by purple color, light blue color, yellow color, black color, red color and green color, respectively.

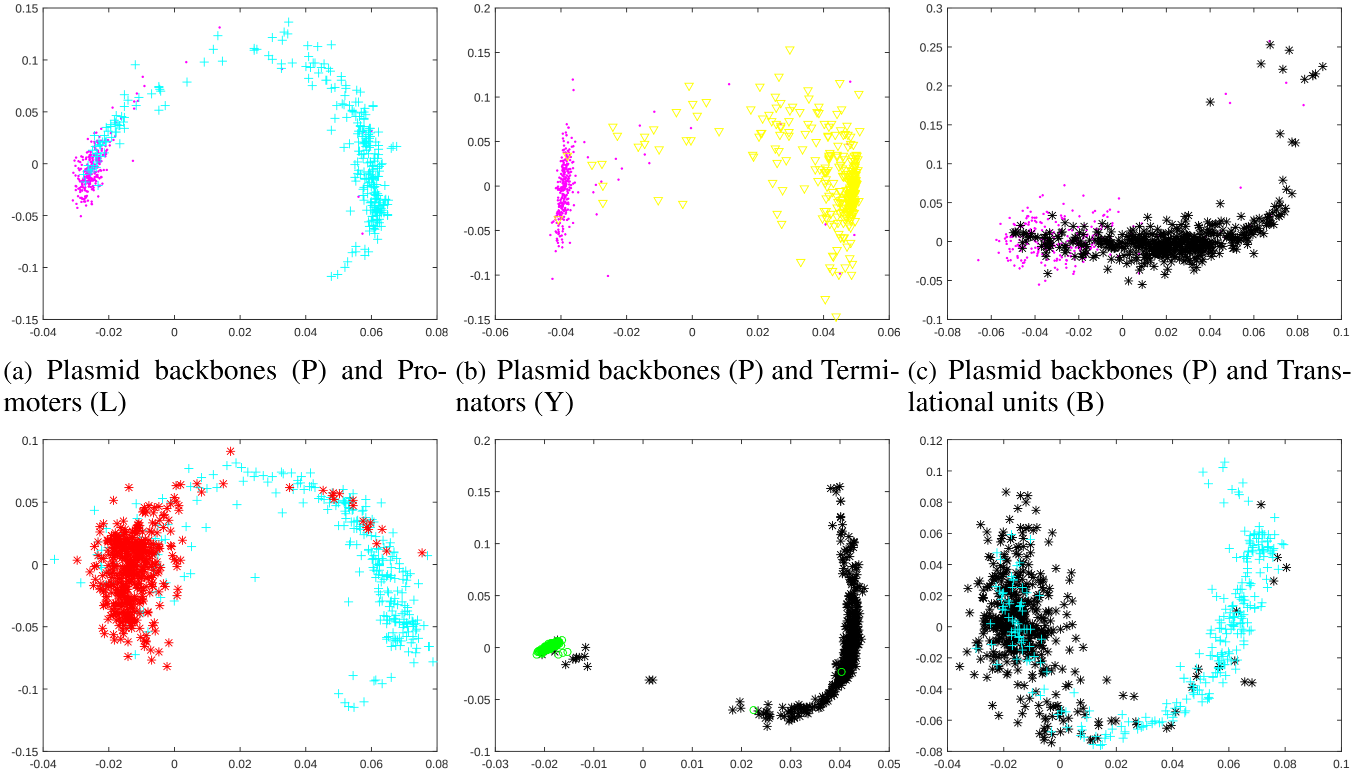

(a) Plasmid backbones (P) and Promoters (L)

(b) Plasmid backbones (P) and Terminators (Y)

(c) Plasmid backbones (P) and Translational units (B)

(d) Promoters (L) and Protein generators (R)

(e) Translational units (B) and Primers (G)

(f) Translational units (B) and Promoters (L)

Figure S2: Dimensionality reduction results for various combinations of Plasmid backbones, Promoters, Terminators, Translational units, Protein generators, Primers by applying Laplacian Eigenmaps algorithm, where these types of biobricks are denoted by purple color, light blue color, yellow color, black color, red color and green color, respectively.

S2 Classification validation

Another way to quantify the dimensionality reduction results is to train a classifier. While K-means clustering algorithm is an unsupervised learning method and analyzes unlabeled data directly, most of classifiers are supervised learning methods and need to train a model with labeled data.

Here we adopt SVM to train a classifier to test classification accuracies. 5-fold experiments are conducted. Table S2 shows the results of 12 combinations of various types of biobricks. The average accuracies for Isomap and Laplacian Eigenmaps are both 0.914. Considering the average clustering accuracies on these 12 groups of data are 0.87 and 0.861, respectively, the classifier achieves better performance than clustering methods. This is reasonable, since the classifier first uses labeled data to train a model, and then validates the performance, while clustering methods conduct the analysis on unlabeled data directly.

Table S2: Classification accuracy comparison of dimensionality reduction results by Isomap and Laplacian Eigenmaps

	Isomap	Laplacian Eigenmaps
Protein Generators and Primer	0.991	0.988
Protein Generators and Protein domains	0.87	0.86
Protein Generators and RBS	0.964	0.959
Primer and Protein domains	0.89	0.904
Protein domains and RBS	0.861	0.866
Primer and RBS	0.841	0.861
Plasmid backbones and Promoters	0.93	0.92
Plasmid backbones and Terminators	0.943	0.967
Plasmid backbones and Translational units	0.895	0.894
Promoters and Protein generators	0.931	0.912
Translational units and Primers	0.927	0.943
Translational units and Promoters	0.924	0.894
